# Supplementary material for: Characterizing the reproductive transcriptomic correlates of acute dehydration in males in the desert-adapted rodent, Peromyscus eremicus
Source: BMC Genomics. 2017 Jun 23;18:473. doi: 10.1186/s12864-017-3840-1 (PMC5481918; doi:10.1186/s12864-017-3840-1)
Supplement: Supplementary file 1 — Supplemental Data. List of supplemental data files available in DropBox and GitHub. (DOCX 40 kb) [file 12864_2017_3840_MOESM1_ESM.docx]

**Supplemental Files on Dryad (doi:10.5061/dryad.743p5):**

Optimized final un-annotated transcriptome (good.BINPACKER.cdhit.fasta)

Annotated transcriptome (good.BINPACKER.cdhit.fasta.dammit.fasta)

Dammit gff3 file of annotation (good.BINPACKER.cdhit.fasta.dammit.gff3)

Salmon folder including salmon quant outputs for 22 individuals (salmon)

Salmon merged quant file (NEWmergedcounts.txt)

Gene ID by Transcript ID matrix (NEWESTfinalMUS.txt)

Transcripts without matches from edgeR DTE analysis (DTEno-matchBLASTnSequences.md)

Accession ID file for PANTHER: relatively high DRY expression gene list (highENSM.csv)

Accession ID file for PANTHER: relatively high WET expression gene list (lowENSM.csv)

PANTHER GO List results for relatively high DRY expression gene list (highENSMgoLIST.txt)

PANTHER GO List Results for relatively high WET expression gene list (lowENSMgoLIST.txt)

***Markdown files for analyses from GitHub site: <https://github.com/macmanes-lab/testesDGE>

Are also available on Dryad (Folder name: testesDGE-master 2)
