# Supplementary material for: Characterizing the reproductive transcriptomic correlates of acute dehydration in males in the desert-adapted rodent, Peromyscus eremicus
Source: BMC Genomics. 2017 Jun 23;18:473. doi: 10.1186/s12864-017-3840-1 (PMC5481918; doi:10.1186/s12864-017-3840-1)
Supplement: Supplementary file 2 — Testes read data statistics, including sample identification (Mouse ID), number of reads (# Reads), percent reads mapped to transcriptome (% Mapping), and treatment group (TRT). Mouse ID 335 T* is the dataset which was used to assemble the testes transcriptome; therefore, these reads were not used for the differential expression analysis. (DOCX 41 kb) [file 12864_2017_3840_MOESM2_ESM.docx]

Supplemental Table 1: Testes read data statistics, including sample identification (Mouse ID), number of reads (# Reads), percent reads mapped to transcriptome (% Mapping), and treatment group (TRT). Mouse ID 335T* is the dataset which was used to assemble the testes transcriptome; therefore, these reads were not used for the differential expression analysis.

| Mouse ID | # Reads | % Mapping | TRT |
| --- | --- | --- | --- |
| ***335T**** | ***45759114*** | ***85.46*** | ***wet*** |
| 3333T | 15135923 | 82.56 | Wet |
| 2322T | 12584407 | 82.37 | Dry |
| 382T | 14305186 | 83.87 | Dry |
| 381T | 14178847 | 83.23 | Wet |
| 376T | 14588175 | 82.56 | Dry |
| 366T | 13641731 | 82.95 | Wet |
| 349T | 17289781 | 85.93 | Wet |
| 209T | 11724617 | 84.02 | Dry |
| 265T | 11536510 | 84.17 | Dry |
| 383T | 13250034 | 81.46 | Dry |
| 384T | 12152820 | 82.75 | Dry |
| 102T | 11131941 | 84.84 | Wet |
| 400T | 13259393 | 83.98 | Wet |
| 1357T | 20603232 | 82.32 | Wet |
| 1358T | 12240814 | 86.58 | Wet |
| 1359T | 11144962 | 85.54 | Wet |
| 13T | 11075885 | 83.55 | Dry |
| 343T | 9423867 | 83.58 | Dry |
| 344T | 17146134 | 85.36 | Wet |
| 355T | 13948415 | 85.21 | Wet |
| 888T | 18890387 | 86.52 | Dry |
| 999T | 15213425 | 87.02 | Dry |
