# Supplementary figures and images for: Characterizing the reproductive transcriptomic correlates of acute dehydration in males in the desert-adapted rodent, Peromyscus eremicus
Source: BMC Genomics. 2017 Jun 23;18:473. doi: 10.1186/s12864-017-3840-1 (PMC5481918; doi:10.1186/s12864-017-3840-1)

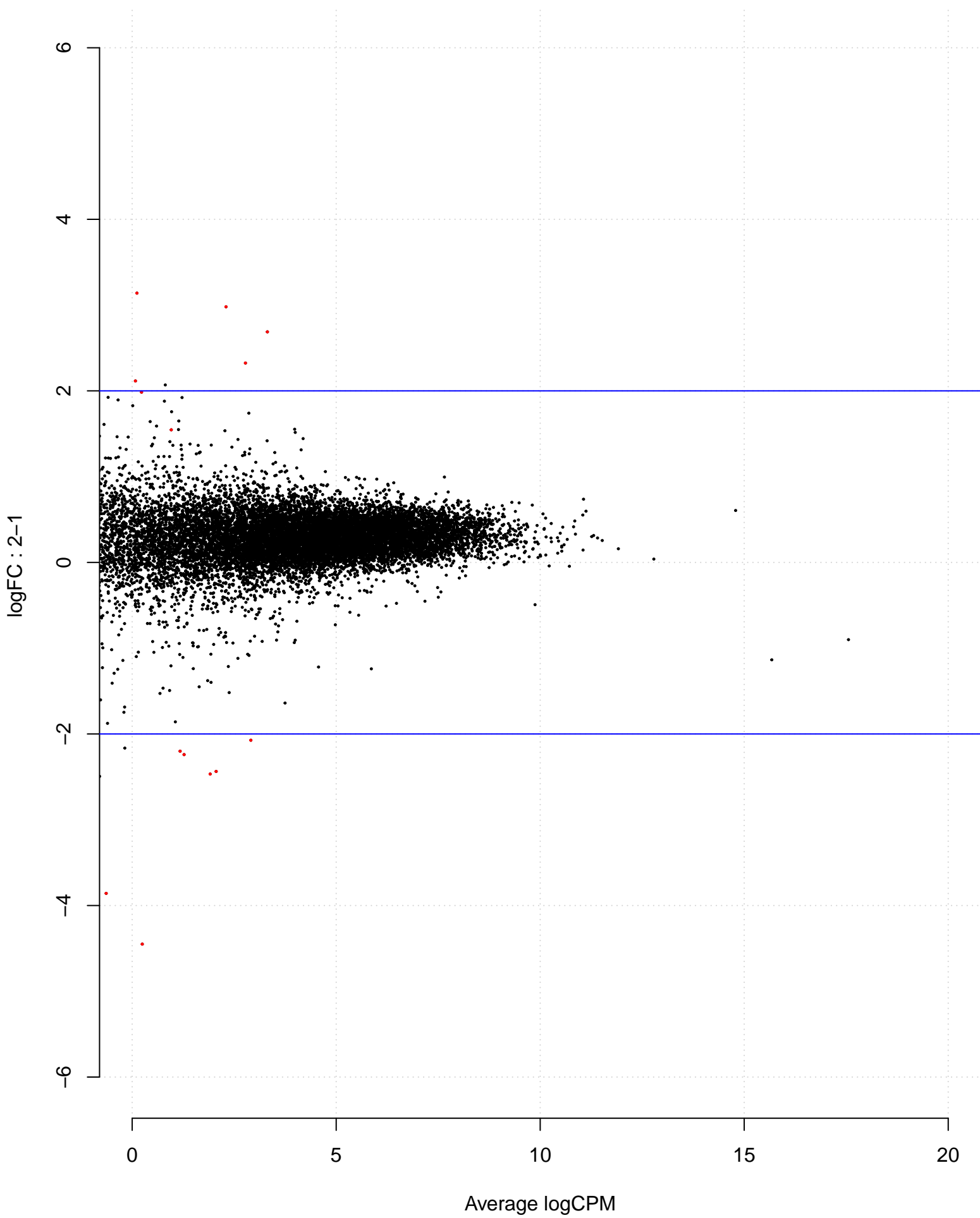

Supplement: Supplementary file 3 — Plot of edgeR determined differentially expressed genes. The 15 significant genes are in red, with positive values indicating increased expression in the DRY group, and negative values depicting increased expression in the WET group. (PDF 760 kb) [file 12864_2017_3840_MOESM3_ESM.pdf]

DE genes, all data, FDR 0.05

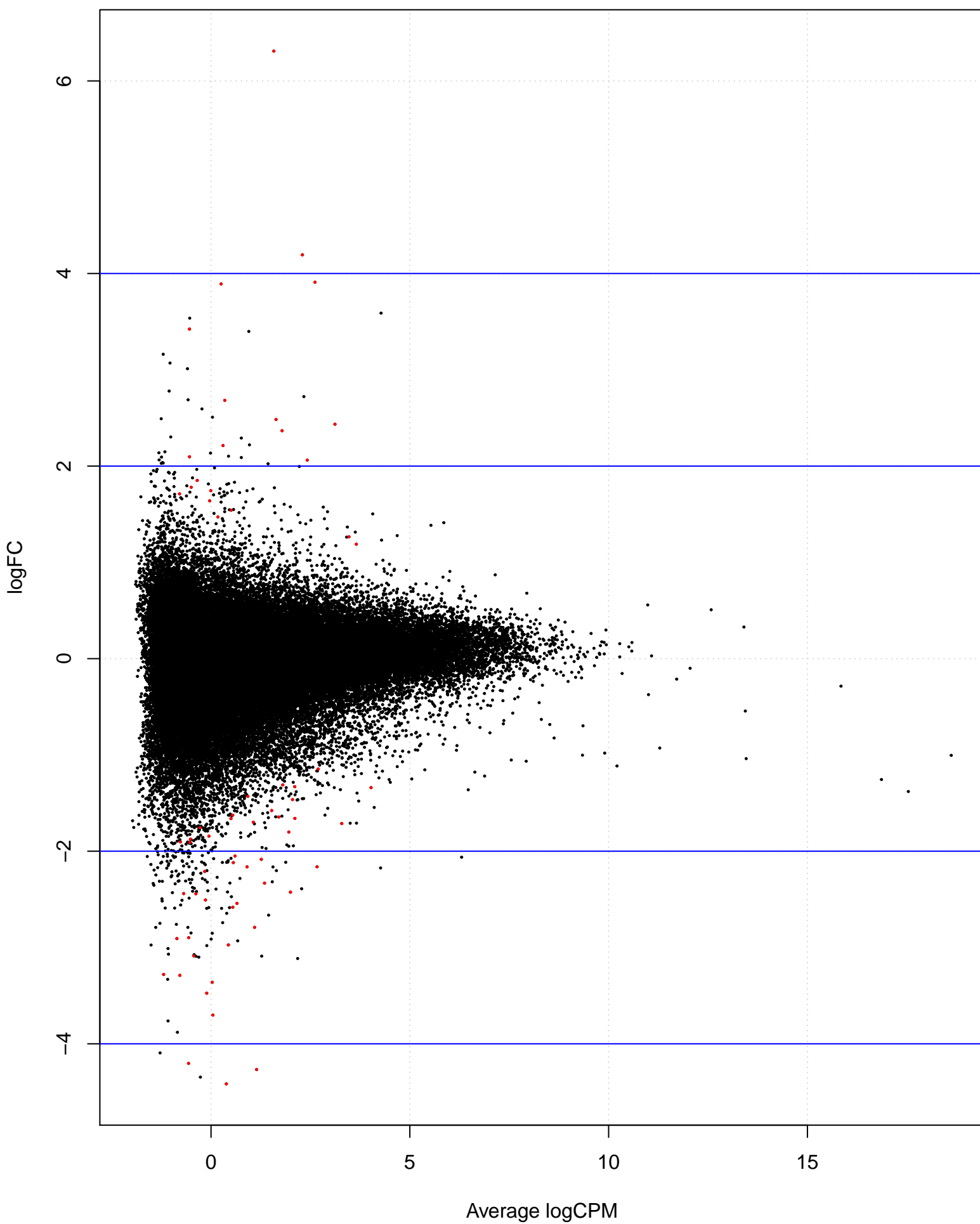

Supplement: Supplementary file 4 — Plot of edgeR determined differentially expressed transcripts. The 66 significant transcripts are in red, with positive values indicating increased expression in the DRY group, and negative values depicting increased expression in the WET group. (PDF 3703 kb) [file 12864_2017_3840_MOESM4_ESM.pdf]

# unshrunk LFC

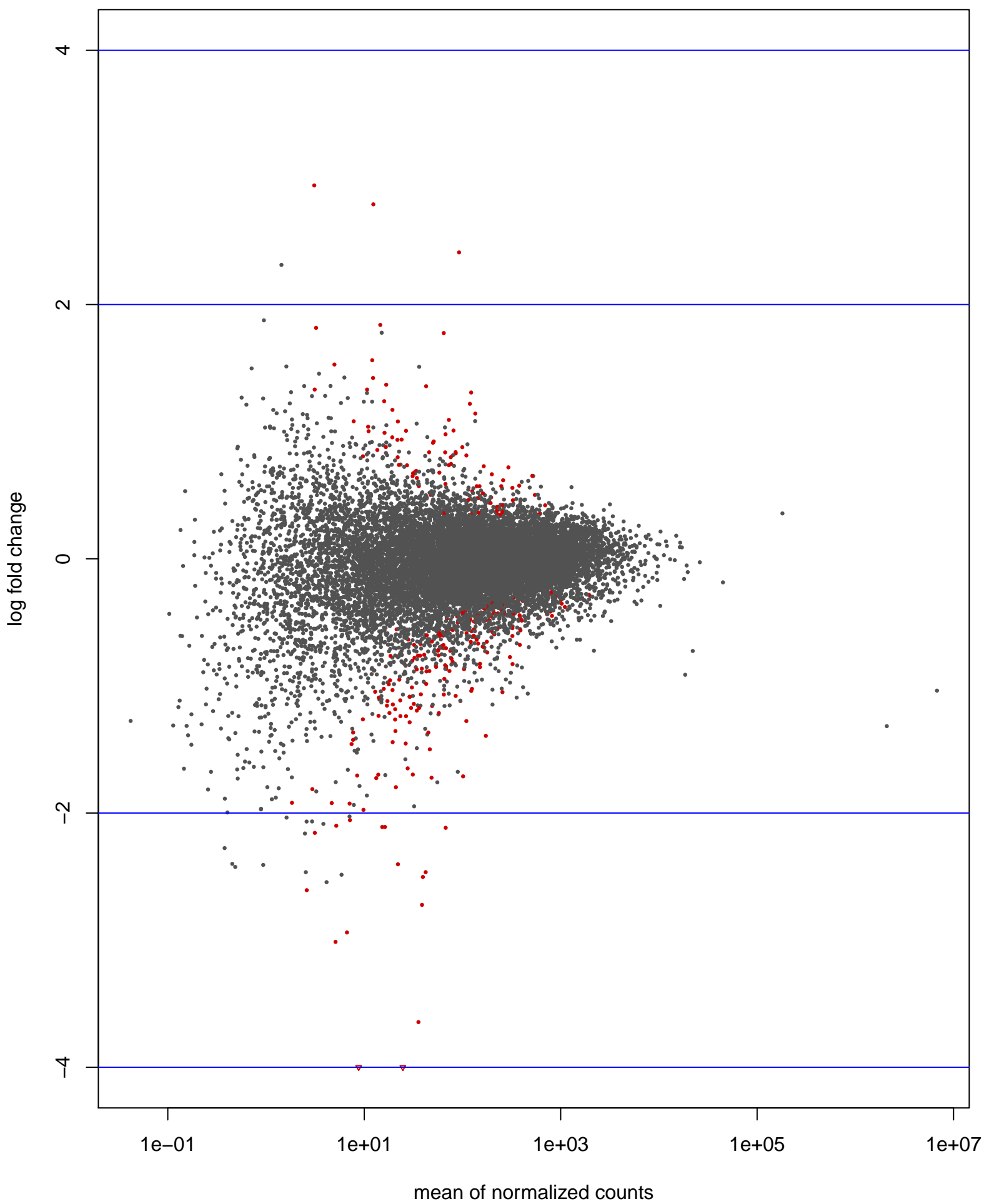

Supplement: Supplementary file 5 — Plot of DESeq2 determined differentially expressed transcripts. The 215 significant transcripts are in red, with positive values indicating increased expression in the DRY group, and negative values depicting increased expression in the WET group. (PDF 829 kb) [file 12864_2017_3840_MOESM5_ESM.pdf]

# Median

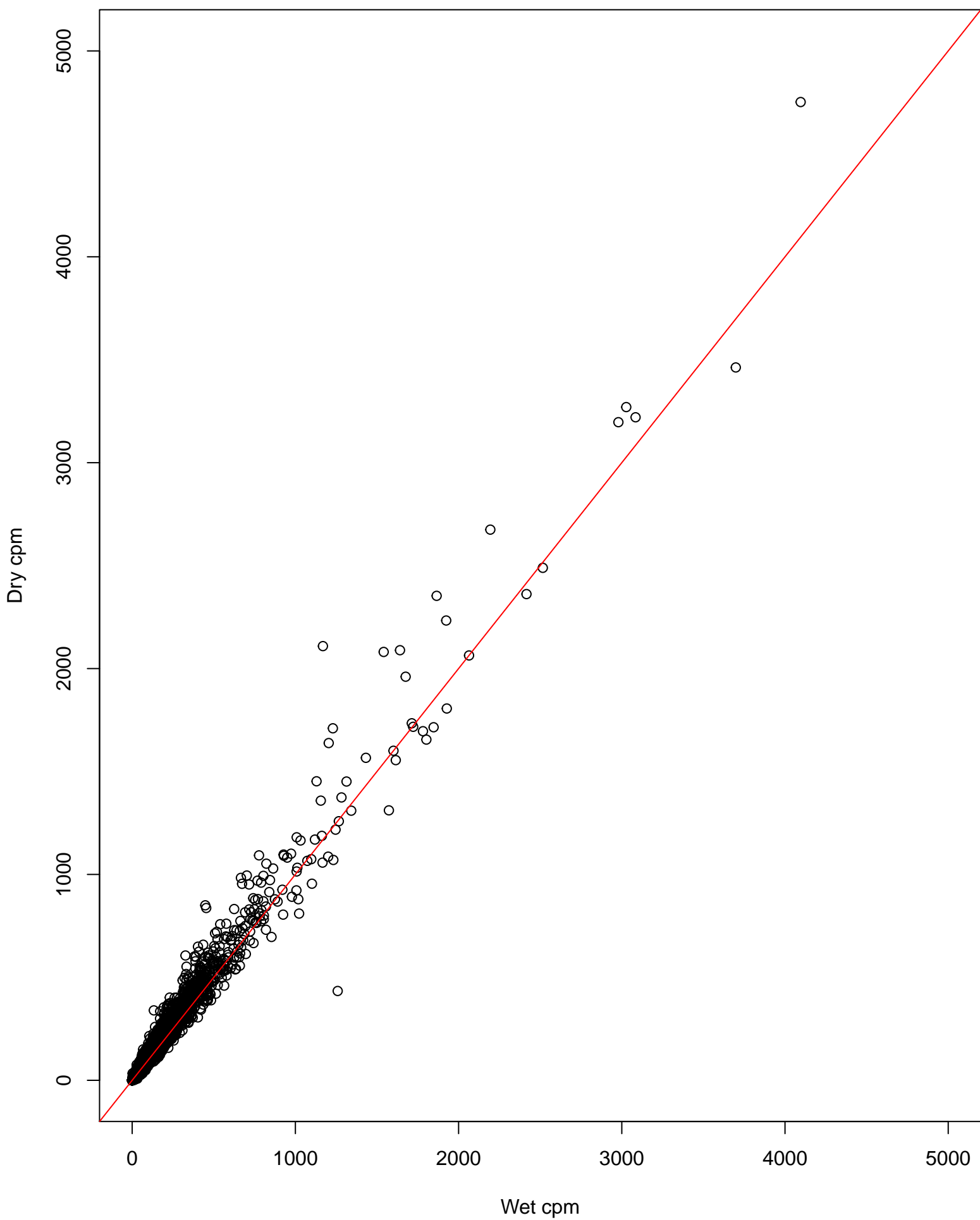

Supplement: Supplementary file 7 — Correlation of edgeR DGE analyzed expression patterns for all individual genes between WET and DRY treatments, as indicated by the correlation between the median values for WET and the median values for DRY normalized cpms (Adj-R2 = 0.616; F(1,14,216) = 151.132; p = 0.00). (PDF 710 kb) [file 12864_2017_3840_MOESM7_ESM.pdf]
