# Supplementary material for: Characterizing the reproductive transcriptomic correlates of acute dehydration in males in the desert-adapted rodent, Peromyscus eremicus
Source: BMC Genomics. 2017 Jun 23;18:473. doi: 10.1186/s12864-017-3840-1 (PMC5481918; doi:10.1186/s12864-017-3840-1)
Supplement: Supplementary file 6 — Significantly differentially expressed genes identified in the three analyses (DGE in edgeR, DTE in edgeR, and DGE in DESeq2) by treatment group in P. eremicus testes. Of the 34 different genes which were more highly expressed in WET mice, six were significant across all three analyses (Gene IDs are italicized). Of the 17 genes which were more highly expressed in DRY mice, three were significant across all three analyses (Gene IDs are italicized). (DOCX 44 kb) [file 12864_2017_3840_MOESM6_ESM.docx]

Supplemental Table 2: Significantly differentially expressed genes identified in the three analyses (DGE in edgeR, DTE in edgeR, and DGE in DESeq2) by treatment group in *P. eremicus* testes. Of the 34 different genes which were more highly expressed in WET mice, six were significant across all three analyses (Gene IDs are italicized). Of the 17 genes which were more highly expressed in DRY mice, three were significant across all three analyses (Gene IDs are italicized).

| **HIGH: WET** | | | |
| --- | --- | --- | --- |
| **Gene ID** | **DGE edgeR** | **DTE edgeR** | **DGE DESeq2** |
| ***Insl3*** | x | X | x |
| ***Ffar4*** | x | X | x |
| ***Slc45a3*** | x | X | x |
| ***Slc38a5*** | x | X | x |
| ***Itgal*** | x | X | x |
| ***Trf*** | x | X | x |
| Slit1 | x | X |  |
| Cpz |  | X | x |
| Tgfb3 |  | X | x |
| Ces1g |  | X |  |
| Ankrd2 |  | X | x |
| Nvl |  | X |  |
| Ogdhl |  | X | x |
| Pfkfb4 |  | X |  |
| Slc33a1 |  | X |  |
| Anxa9 |  | X | x |
| Ddb2 |  | X |  |
| St3gal1 |  | X |  |
| Acsm5 |  | X | x |
| Cyp17a1 |  | X |  |
| Olfml2b |  | X | x |
| Pf4 |  | X |  |
| Nptx2 |  | X | x |
| Dnah6 |  | X |  |
| Sbpl |  | X | x |
| Adcy6 |  | X |  |
| Gm5424 |  | X | x |
| Mbp |  | X |  |
| Fbxo2 |  | X |  |
| Mycl |  | X |  |
| Eci1 |  | X |  |
| Capn12 |  | X |  |
| Col6a1 |  | X |  |
| Gpr55 |  | X |  |
| **HIGH: DRY** | | | |
| **Gene ID** | **DGE edgeR** | **DTE edgeR** | **DGE DESeq2** |
| ***Rin2*** | x | X | x |
| ***Igfbp3*** | x | X | x |
| ***Ctgf*** | x | X | x |
| Cyp2e1 | x |  |  |
| Fmo2 | x |  |  |
| Tnfrsf21 | x |  |  |
| Cyp2f2 | x |  |  |
| Dennd2d | x |  |  |
| Nedd1 |  | X |  |
| Wdr83 |  | X |  |
| Gpx4 |  | X |  |
| Asah1 |  | X |  |
| Adm |  | X |  |
| Tmem108 |  | X | x |
| Nkx3-1 |  | X |  |
| Ybx1 |  | X |  |
| Arfgef2 |  | X |  |
