# Supplementary material for: Characterizing the reproductive transcriptomic correlates of acute dehydration in males in the desert-adapted rodent, Peromyscus eremicus
Source: BMC Genomics. 2017 Jun 23;18:473. doi: 10.1186/s12864-017-3840-1 (PMC5481918; doi:10.1186/s12864-017-3840-1)
Supplement: Supplementary file 8 — PANTHER GO-Slim Molecular Function results for the relatively high DRY expression gene list and the relatively high WET expression gene list. Number of genes (# WET; # DRY), percent of gene hits against total number of function hits (% WET; % DRY). (DOCX 40 kb) [file 12864_2017_3840_MOESM8_ESM.docx]

Supplemental Table 3: PANTHER GO-Slim Molecular Function results for the relatively high DRY expression gene list and the relatively high WET expression gene list. Number of genes (# WET ; # DRY), percent of gene hits against total number of function hits (% WET; % DRY).

| **PANTHER GO-Slim Molecular Function** | **# WET** | **% WET** | **# DRY** | **% DRY** |
| --- | --- | --- | --- | --- |
| transporter activity (GO:0005215) | 82 | 8.4% | 153 | 6.6% |
| translation regulator activity (GO:0045182) | 7 | 0.7% | 14 | 0.6% |
| catalytic activity (GO:0003824) | 335 | 34.3% | 871 | 37.8% |
| channel regulator activity (GO:0016247) | 2 | 0.2% | 6 | 0.3% |
| receptor activity (GO:0004872) | 75 | 7.7% | 176 | 7.6% |
| signal transducer activity (GO:0004871) | 26 | 2.7% | 92 | 4.0% |
| antioxidant activity (GO:0016209) | 5 | 0.5% | 5 | 0.2% |
| structural molecule activity (GO:0005198) | 88 | 9.0% | 139 | 6.0% |
| binding (GO:0005488) | 356 | 36.5% | 848 | 36.8% |
