# Supplementary material for: Characterizing the reproductive transcriptomic correlates of acute dehydration in males in the desert-adapted rodent, Peromyscus eremicus
Source: BMC Genomics. 2017 Jun 23;18:473. doi: 10.1186/s12864-017-3840-1 (PMC5481918; doi:10.1186/s12864-017-3840-1)
Supplement: Supplementary file 9 — PANTHER Overrepresentation TEST GO-Slim Biological Process results for the relatively high DRY expression gene list analyzed against the high WET expression gene list as the reference list. This test reports significantly over- and under-representation of the high DRY expression gene list for multiple GO categories. For each GO term, we report the number of GO matches in both gene lists (# WET; # DRY) compared to the expected number of DRY matches (DRY EXP), the over- (+) or under- (−) representation, fold enrichment (Fold Enr), and significance (P-value). (DOCX 44 kb) [file 12864_2017_3840_MOESM9_ESM.docx]

Supplemental Table 4: PANTHER Overrepresentation TEST GO-Slim Biological Process results for the relatively high DRY expression gene list analyzed against the high WET expression gene list as the reference list. This test reports significantly over- and under-representation of the high DRY gene expression gene list for multiple GO categories. For each GO term, we report the number of GO matches in both gene lists (# WET ; # DRY) compared to the expected number of DRY matches (DRY EXP), the over- (+) or under- (-) representation, fold enrichment (Fold Enr), and significance (P-value).

| **PANTHER GO-Slim**  **Biological Process** | **# WET** | **# DRY** | **DRY EXP** | **o / u** | **Fold Enr** | **P-value** |
| --- | --- | --- | --- | --- | --- | --- |
| transcription initiation from RNA POL II promoter (GO:0006367) | 1 | 13 | 2.42 | + | 5.37 | 3.15E-04 |
| vitamin metabolic process (GO:0006766) | 1 | 12 | 2.42 | + | 4.96 | 1.72E-03 |
| induction of apoptosis  (GO:0006917) | 1 | 11 | 2.42 | + | 4.55 | 8.72E-03 |
| cell proliferation  (GO:0008283) | 2 | 21 | 4.84 | + | 4.34 | 8.74E-06 |
| response to endogenous stimulus (GO:0009719) | 2 | 21 | 4.84 | + | 4.34 | 8.74E-06 |
| nuclear transport  (GO:0051169) | 4 | 28 | 9.68 | + | 2.89 | 2.23E-04 |
| polysaccharide metabolic process (GO:0005976) | 6 | 40 | 14.52 | + | 2.75 | 4.86E-06 |
| regulation of catalytic activity (GO:0050790) | 5 | 33 | 12.1 | + | 2.73 | 9.51E-05 |
| mRNA processing  (GO:0006397) | 12 | 52 | 29.04 | + | 1.79 | 1.37E-02 |
| vesicle-mediated transport (GO:0016192) | 43 | 147 | 104.07 | + | 1.41 | 6.02E-03 |
| RNA metabolic process (GO:0016070) | 122 | 367 | 295.27 | + | 1.24 | 2.50E-03 |
| Unclassified  (UNCLASSIFIED) | 579 | 1420 | 1401.31 | + | 1.01 | 0.00E+00 |
| cellular component organization or biogenesis (GO:0071840) | 155 | 299 | 375.14 | - | 0.8 | 1.88E-03 |
| cellular component organization (GO:0016043) | 141 | 267 | 341.25 | - | 0.78 | 1.29E-03 |
| organelle organization  (GO:0006996) | 89 | 143 | 215.4 | - | 0.66 | 7.92E-06 |
| mitosis  (GO:0007067) | 37 | 58 | 89.55 | - | 0.65 | 3.98E-02 |
| DNA metabolic process (GO:0006259) | 35 | 52 | 84.71 | - | 0.61 | 1.44E-02 |
| anion transport  (GO:0006820) | 23 | 31 | 55.67 | - | 0.56 | 3.94E-02 |
| chromatin organization (GO:0006325) | 54 | 56 | 130.69 | - | 0.43 | 1.11E-11 |
| muscle contraction  (GO:0006936) | 14 | 13 | 33.88 | - | 0.38 | 6.75E-03 |
| monosaccharide metabolic process (GO:0005996) | 14 | 13 | 33.88 | - | 0.38 | 6.75E-03 |
| regulation of gene expression, epigenetic (GO:0040029) | 14 | 12 | 33.88 | - | 0.35 | 2.46E-03 |
| protein acetylation  (GO:0006473) | 7 | 4 | 16.94 | - | 0.24 | 3.58E-02 |
| oxidative phosphorylation (GO:0006119) | 14 | 5 | 33.88 | - | < 0.2 | 1.40E-07 |
| chromatin assembly  (GO:0031497) | 10 | 1 | 24.2 | - | < 0.2 | 1.36E-07 |
